# *In Vitro* and *in Vivo* Evaluation of Mutations in the NS Region of Lineage 2 West Nile Virus Associated with Neuroinvasiveness in a Mammalian Model

**DOI:** 10.3390/v8020049

**Published:** 2016-02-19

**Authors:** Katalin Szentpáli-Gavallér, Stephanie M. Lim, László Dencső, Krisztián Bányai, Penelope Koraka, Albert D.M.E. Osterhaus, Byron E.E. Martina, Tamás Bakonyi, Ádám Bálint

**Affiliations:** 1 Veterinary Diagnostic Directorate, National Food Chain Safety Office, H-1143, Budapest, Hungary; balintad@nebih.gov.hu (A.B.); dencsol@nebih.gov.hu (L.D.); 2 Viroscience Laboratory, Erasmus Medical Centre, 3015CN, Rotterdam, The Netherlands; s.lim@erasmusmc.nl (S.M.L.); p.koraka@erasmusmc.nl (P.K.); a.osterhaus@erasmusmc.nl (A.D.M.E.O.); b.martina@erasmusmc.nl (B.E.E.M.); 3 Institute for Veterinary Medical Research, Centre for Agricultural Research, Hungarian Academy of Sciences, H-1143, Budapest, Hungary; bkrota@hotmail.com; 4 Department of Microbiology and Infectious Diseases, Faculty of Veterinary Science, Szent István University, H-1143, Budapest, Hungary; Bakonyi.Tamas@aotk.szie.hu; 5 Viral Zoonoses, Emerging and Vector-Borne Infections Group, Institute of Virology, University of Veterinary Medicine, A-1210, Vienna, Austria

**Keywords:** West Nile virus, lineage 2, virulence markers, infectious clone

## Abstract

West Nile virus (WNV) strains may differ significantly in neuroinvasiveness in vertebrate hosts. In contrast to genetic lineage 1 WNVs, molecular determinants of pathogenic lineage 2 strains have not been experimentally confirmed so far. A full-length infectious clone of a neurovirulent WNV lineage 2 strain (578/10; Central Europe) was generated and amino acid substitutions that have been shown to attenuate lineage 1 WNVs were introduced into the nonstructural proteins (NS1 (P250L), NS2A (A30P), NS3 (P249H) NS4B (P38G, C102S, E249G)). The mouse neuroinvasive phenotype of each mutant virus was examined following intraperitoneal inoculation of C57BL/6 mice. Only the NS1-P250L mutation was associated with a significant attenuation of virulence in mice compared to the wild-type. Multiplication kinetics in cell culture revealed significantly lower infectious virus titres for the NS1 mutant compared to the wild-type, as well as significantly lower amounts of positive and negative stranded RNA.

## 1. Introduction

West Nile virus (WNV) is one of the most widely distributed members of the genus *Flavivirus* within the family *Flaviviridae*. The virus can cause general, febrile and severe encephalitic disease in humans and a number of animal species. It possesses a capped and non-polyadenylated positive-sense, single-stranded RNA genome, approximately 11 kb in length, which contains a single open reading frame (ORF) encoding a polyprotein precursor that is co- and post-translationally processed by viral and host cell proteases to three structural proteins (capsid protein C, pre-membrane protein prM/M, envelope protein E) and seven non-structural (NS) proteins (glycoprotein NS1, NS2A, protease cofactor NS2B, protease and helicase NS3, NS4A, NS4B and polymerase NS5). 

WNV strains have been divided into at least eight distinct genetic lineages [1,2,3]. The first WNV strain, isolated in Uganda in 1937 [4], belongs to lineage 2 and this lineage has been confined to sub-Saharan Africa and Madagascar [5]. Until the early 1990s, WNV infections beyond Africa were caused by lineage 1 WNV strains that caused mainly mild clinical symptoms with sporadic encephalitis in humans. Lineage 1 WNV as the causative agent of lethal encephalitis was reported in 1994, in Algeria, followed by a high number of neuroinvasive cases in Romania, in 1996, and more frequent encephalitis cases in several other Eastern and Western European countries [6]. Moreover, in 1999, WNV emerged in the continental United States and rapidly spread throughout North America and also into South America, causing increased mortality among humans and animals as well [7,8,9]. 

Lineage 2 strains did not appear to be as pathogenic as lineage 1 isolates, as they generally caused no deaths, and only mild clinical signs of infection [10]. The first proven case of a lineage 2 virus infection with fatal outcome outside of Africa was detected in a goshawk (*Accipiter gentilis*) in Hungary, in 2004 [11,12]. This exotic WNV lineage 2 strain caused subsequent infections, illnesses and deaths in wild birds, horses and humans [11,12,13], and rapidly spread to Austria [14,15,16], Greece [17,18] and Italy [19,20]. Based on phylogenetic reconstruction, lineage 2 viruses isolated in Russia [21,22] and Romania [23] cluster together, but are distinct from the Hungarian strain [16]. 

Several studies have aimed at identifying the molecular markers of virulence in lineage 1 WNV strains [24,25,26,27]. A number of lineage 1 viruses of moderate virulence in mice and exhibiting inefficient growth in cell culture have been identified so far [28]. Reverse genetic systems were established to investigate nucleotide and amino acid alterations in the WNV lineage 1 genome that led to decreased neurovirulence and neuroinvasiveness in mice [29,30]. Mutations in the NS protein genes, such as *NS1* [31], *NS2A* [24,26,32], *NS3* [33], *NS4B* [30,34,35,36] and *NS5* [34] ,were found to be associated with the attenuation of these viruses. 

In contrast to lineage 1 viruses, molecular determinants of pathogenic WNV lineage 2 strains have not been confirmed experimentally. Virulence markers have only been identified *in silico* by analyzing and comparing full genome sequences of highly or less neuroinvasive lineage 2 strains [37], or by comparing those that have emerged and increased in virulence over time (1937-2011) [38]. The aim of the present study was to investigate the *in vitro* (in cell culture) and *in vivo* (in a mouse model) effects of selected nucleotide substitutions of the NS protein coding genes, known to be attenuating for lineage 1, in a neurovirulent WNV lineage 2 strain (578/10) isolated in Central Europe with the help of reverse genetic methods and site-specific mutagenesis.

## 2. Materials and Methods 

### 2.1. Cells and Viruses

Transfection was carried out on baby hamster kidney 21 (BHK-21) cells (ATCC^®^: CCL-10^TM^) cultured in Dulbecco’s Modified Eagle’s Medium (DMEM) supplemented with 5% foetal bovine serum (FBS). Virus stocks were propagated and titrated on Vero E6 cells (ATCC^®^: CRL-1586^TM^) cultured in DMEM supplemented with 0.75% sodium bicarbonate, 10 mM hepes buffer and 10% FBS. All media was supplemented with antibiotics (100 U penicillin, 100 µg/mL streptomycin) and 2 mM L-glutamine. The WNV-578/10 strain (GenBank accession number: KC496015) was originally isolated in Hungary from a horse showing clinical signs of encephalitis, which had died in 2010.

### 2.2. RNA Extraction and cDNA Synthesis 

Total RNA was extracted from virus pellet using the QIAamp Viral RNA Mini Kit (QIAGEN, Hilden, Germany) following the manufacturer’s instructions. To obtain long cDNA copies of the viral genome, reverse transcription was performed using the high fidelity SuperScript III First-Strand Synthesis System (Invitrogen, Carlsbad, CA, USA) and gene-specific reverse primers (Table 1). In the first step, 0.5 µL of each primer (10 µM) and 1 µL RNA in 7.5 µL RNase-free water were heated to 65 °C for 5 minutes and then cooled down to 4 °C for 10 minutes. In the second step, 11 µL reaction mixture (2 µL reaction buffer, 4 µL MgCl_2_, 2 µL dithiothreitol (DTT), 1 µL RNase OUT inhibitor, 1 µL SSIII enzyme and 1 µL dNTP mixture) were added and after 30 minutes at room temperature and the final 20-µL reaction mixture was heated to 50 °C for 90 minutes, followed by 85 °C for 5 minutes, and finally cooled down to 4 °C. Before amplification, cDNA was treated with RNase H (1 µL RNase H was added to 20 µL of the above-mentioned solution and was heated to 37 °C for 20 minutes).

### 2.3. Sequence Analysis

The complete genome of WNV-578/10 was determined by sequencing of overlapping PCR fragments amplified with Phusion Hot Start High-Fidelity DNA Polymerase (Finnzymes, Espoo, Finland). Amplification products were directly sequenced with the ABI Prism BigDye Terminator V3.1 Cycle Sequencing Kit (Applied Biosystems, Foster City, CA, USA) and an automated ABI 377 DNA Sequencer (Applied Biosystems). Sequence assembly and comparison were performed with SeqMan and MegAlign programs (Lasergene, Madison, WI, USA), respectively. The full-length WNV clone was sequenced with the ABI Prism BigDye Terminator V3.1 Cycle Sequencing Kit (Applied Biosystems) and an ABI Prism 310 genetic analyzer (Applied Biosystems), as described in Bakonyi *et al.*, 2004 [39]. 

Full genome sequencing of WNV clones with the incorporated modifications was carried out by using semiconductor sequencing technology. Briefly, overlapping PCR fragments were generated as described above. Equimolar amounts of the amplicons from each clone were used for preparation of Ion Torrent compatible libraries. Clonal amplification of library DNA (with 200 bp inserts) by emulsion PCR on an Ion One Touch v2 (Life Technologies, Thermo Fisher Scientific, Waltham, MA, United States) equipment was followed by enrichment on an Ion OneTouch ES pipetting robot, and then by sequencing on a 316 Chip using Ion Torrent PGM (Life Technologies). Raw reads were curated and then consensus genomic sequences were assembled using the CLC Genomics Workbench version 7 (www.clcbio.com).

### 2.4. Plasmids and Bacteria Strains

Plasmid pBeloBAC TGE [40] was kindly provided by L. Enjuanes (*Centro Nacional de Biotecnologia*, Department of Molecular and Cell Biology, Madrid, Spain). The plasmid was propagated in Max Efficiency DH10B competent *Escherichia coli* cells (Invitrogen) that were transformed by heat shock according to the manufacturer’s instructions. Modifications, such as deletion of the *XhoI* site downstream of the 3’ accessory sequences and insertion of a multiple cloning site containing *SfiI*, *SfoI*, *BstBI*, *ClaI*, *BamHI*, *XhoI*, *AsiSI*, *AvrII* and *SacII*, were described earlier [41]. For large-scale DNA preparation, the BAC vector and recombinant BACs were isolated with the QIAGEN Large Construct Kit (QIAGEN) according to the manufacturer’s specifications. 

### 2.5. Generation of the Full-Length Clone of WNV-578/10 

**Figure 1:**
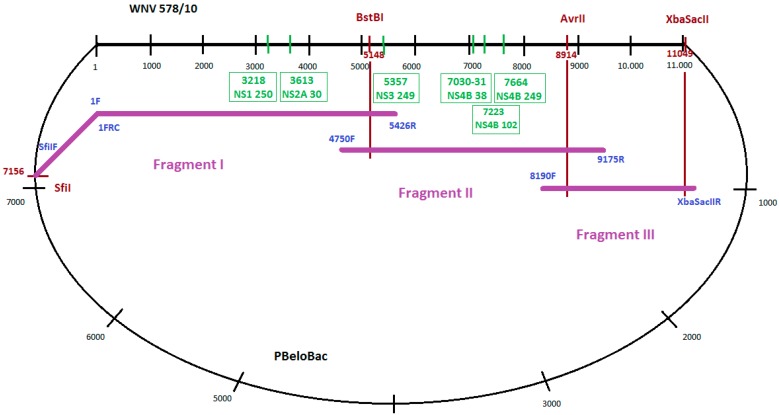
Cloning strategy for the WNV-578/10 genome and positions of the generated mutations. Black numbers represent nucleotide positions in WNV genome/PBeloBac plasmid genome. Red numbers indicate nucleotide positions of restriction enzyme cleavage sites in WNV genome. Green numbers in boxes represent nucleotide positions of inserted mutations. Names of used primers are in blue.

To amplify long, overlapping PCR products, ranging between 1 and 5.5 kb covering the whole genome in three fragments (Fragment I, II and III), PCRs were performed using genome-specific primers (Figure 1). Long-range PCR assays were implemented using the Phusion Hot Start High-Fidelity DNA Polymerase (Finnzymes). The construction of Fragment I was performed in two steps. First, the CMV promoter sequence from the PBeloBAC (with primers 5’SfiIF and 1FRC), as well as the 5’ side of 5426 nucleotides of WNV-578/10 strain (with primers 1F and 5426R), were amplified. Primers 1F and 1FRC were reverse complements. In the second step that joined together the two amplicons, a fusion PCR utilizing the overlapping sequences of the above-mentioned primers was performed with Phusion Hot Start High-Fidelity DNA Polymerase (Finnzymes). Fragment II was amplified by using primers 4750F and 9175R. For amplification of Fragment III, a specific reverse primer containing two extra restriction sites (*XbaI* and *SacII*) in addition to the 3’ conservative end of WNV-578/10 strain (3’XbaSacIIR) and forward primer 8190F was used. Sequences of all the primers used to generate the full-length clone are listed in Table 1. All fragments were separated and purified from 0.8% agarose gel using the QIAquick Gel Extraction kit (Qiagen). 

Restriction enzyme cleavage and cloning steps were performed according to standard protocols [42]. Restriction endonucleases and DNA modifying enzymes were purchased from Fermentas (Vilnius, Lithuania) and New England Biolabs (Beverly, MA, USA). The cDNA fragments were ligated with T4 DNA Ligase (Invitrogen Life Technologies). After the digestion of pBeloBac and fragment III with *AvrII* and *SacII* restriction endonucleases, fragment III was cloned into the pBeloBac to obtain WNV-3. Fragment II was digested with *BstBI* and *AvrII* restriction endonucleases, and was cloned into WNV-3 to get WNV-3-2. Fragment I was digested with *SfiI* and *BstBI* restriction endonucleases, and was cloned into WNV-3-2 to obtain the final full-length clone pWNV-578/10. All recombinant plasmids were stable in bacteria, and no toxicity was observed. All the sequences of molecular constructs were confirmed by restriction endonuclease pattern analysis and PCRs.

### 2.6. Generation of Mutant Full-Length WNV Clones

Point mutations were inserted in the genome of WNV using PCR-based mutagenesis utilizing specific oligonucleotides (forward and reverse, complementary oligos) containing the altered nucleotides (Table 2). Depending on the position of the mutation in the genome, new fragments I or II were synthesized using the mutated complementary oligos in fusion PCRs. In the next step, the original fragment I or fragment II was removed from the pWNV-578/10 clone and was replaced with the newly synthesized fragment I or fragment II, containing the respective mutations in the pWNV-578/10 clone. The generated mutations were as follows: C3218T in NS1 protein gene (P250L), G3613C in NS2A protein gene (A30P), C5357A in NS3 protein gene (P249H), and three mutations in the NS4B protein gene: CC7030-31GG (P38G), G7223C (C102S) and A7664G (E249G) (Figure 1). 

### 2.7. Transfection and Recovery of the Recombinant Viruses from the cDNA Clones

Before transcription, the CMV-WNV clone was double digested with *SfiI* and *XbaI* restriction endonucleases. To remove the single-stranded nucleotide overhang generated by the digestion, Mung Bean Nuclease (New England Biolabs) was used. In order to get the purified expression cassette, pieces from digestion were separated on a 0.8% agarose gel and the approximately 12 kb cassette was purified with QIAquick Gel Extraction Kit (QIAGEN). Four micrograms of double-stranded cDNA was transfected into BHK-21 cells using Turbofect *in vitro* Transfection Reagent (Thermo Scientific, Waltham, MA USA). Briefly, 4 µg of plasmid DNA were transfected into an approximately 60% confluent monolayer of BHK-21 cells on a 6-well culture plate after a 20 minute incubation period with the Turbofect reagent. The plates were incubated for 4 h at 37 °C, then the supernatant was removed, and fresh DMEM (Sigma-Aldrich, Saint Louis, MO, USA) supplemented with 10% FBS was added. The cells were incubated at 37 °C for 48–96 h until a visible cytopathic effect (CPE) appeared. At this time point, supernatant was harvested, clarified by centrifugation at 1000 × g for 5 min, and stored at –80 °C. To increase infectious virus titres for use in *in vitro* and *in vivo* experiments, virus stocks of the recombinant wild type (WT) and mutant viruses were incubated on 80% monolayers of Vero E6 cells in cell culture flasks. At the point of maximum CPE (4–5 days after inoculation), supernatants were harvested, clarified by centrifugation at 1000 × g for 5 min, and stored at –80 °C. Infectious titres of the virus stocks were determined by log_10_ titration on Vero E6 cells and calculating the TCID_50_ using the Spearman–Kärber method [43,44] after determination of CPE 5 days p.i. 

Subsequent full genome sequencing of each stock was performed by semiconductor sequencing as detailed in Section 2.3.

### 2.8. Multiplication Curves in Cell Culture

Growth curves of the wild-type virus and the mutated clones were generated in Vero E6 cells at 37 °C in two independent experiments. Vero E6 cells were seeded at 10^4^ cells/well in flat-bottom 96-well plates, incubated overnight at 37 °C and 80% monolayers were infected in triplicate with 100 µL of virus diluted in serum-free DMEM at multiplicity of infection (MOI) 0.1. Cells were incubated for 1 h at 37 °C and subsequently washed three times with serum-free medium before adding 120 µL of DMEM supplemented with 10% FCS. At 0, 12, 24, 36, 48, 72 and 96 h post-infection (p.i.), supernatants were removed and 70 µL was frozen at –80 °C until used for infectious virus titre determination, while the remaining 50 µL was added to 350 µL of lysis buffer (Roche Diagnostics, Almere, The Netherlands) for RNA extraction. Cells were also collected for RNA extraction in 50 µL of lysis buffer at time points 0, 12, 36 and 48 h p.i. Harvested supernatant infectious virus titres were determined by log_10_ titration on Vero E6 cells as described previously.

### 2.9. Quantitation of Viral RNA Titres

In order to quantify viral RNA copies in the supernatant and cells, RNA was extracted using the MagNA Pure LC Total Nucleic Acid Isolation Kit (Roche Diagnostics) and an automated nucleic acid robotic workstation (Roche Diagnostics) according to the manufacturer’s instructions. RNA was eluted in 50 µL of elution buffer and used immediately in quantitative real-time PCR (qRT-PCR). Within *in vivo* experiments, viral RNA copies in half-brains were quantified after weighing and homogenization using a metal bead in DMEM containing antibiotics (100 U penicillin, 100 µg/mL streptomycin) using a tissue homogenizer. One hundred microliters of tissue homogenate was added to 400 µL of lysis buffer (Roche Diagnostics) and RNA was extracted as indicated above. RNA copy numbers were determined in a strand-specific qRT-PCR assay [45] using a standard curve of *in vitro* transcribed RNA of positive and negative strand of known quantities, which were generated from a plasmid (pCR^®^4-TOPO^®^; Invitrogen, Breda, The Netherlands) containing the sequence of the 3’UTR of WNV. Plasmid was linearized and run-off transcripts were generated using the Ambion^®^ MaxiScript T7/T3 kit (Invitrogen). The positive sense RNA was transcribed using the T3 polymerase and the negative sense RNA using the T7 polymerase. The product was digested with DNase to remove residual DNA and cleaned-up using the High Pure RNA Isolation kit (Roche Diagnostics). *In vitro* transcribed RNA was diluted to a concentration at which DNA was no longer detected.

### 2.10. Mouse Virulence Studies

Six-week-old female C57BL/6 mice (Harlan Laboratories B.V., Venray, The Netherlands) were inoculated intraperitoneally (i.p.) with average doses of 10 and 10^4^ TCID_50_ per mouse of wild-type infectious clone-derived CMV-WNV as well as the mutant viruses (n = 8 per clone, per dose; Table 3). Mice were observed twice daily and were euthanized by cervical dislocation under isoflurane anesthesia when humane endpoints were reached (immobility and paralysis), after which brains were immediately collected for further processing. At 14 days p.i., the end-point for the survival experiment was reached and all remaining mice were euthanized and brains and kidneys were collected from those that had been infected with the highest viral dose. Mice were maintained in specific pathogen-free conditions, had a 12-hour day-night cycle and were fed *ad libitum*. Animal experiments were approved by the Animal Ethics Committee of Erasmus Medical Centre and carried out under protocol number 122-13-19.

### 2.11. Statistical Analysis

Data analysis was performed using GraphPad Prism v5 software statistical analysis. The ANOVA test was used for the comparison of more than two groups. Statistical significance between individual groups was determined using the Mann-Whitney *U* test, and statistical significance was accepted at *p* ≤ 0.05.

## 3. Results

### 3.1. Comparison of the Genome Sequences of WNV Lineage 1 (NY99) and Lineage 2 (578/10) Strains

The complete genome sequence of the 578/10 strain (GenBank accession number KC496015) exhibits 2236 nucleotide (nt) differences (21%) compared to the WNV NY99 strain (accession number AF202541). The nucleotide substitutions are distributed fairly equally within the genome (5’UTR: 0 substitutions (0%), C protein gene: 59 substitutions (16%), preM-M protein gene: 102 substitutions (21%), E protein gene: 309 substitutions (21%), NS1 protein gene: 227 substitutions (22%), NS2A protein gene: 153 substitutions (23%), NS2B protein gene: 82 substitutions (22%), NS3 protein gene: 382 substitutions (21%), NS4A protein gene: 80 substitutions (22%), 2K gene: 13 substitutions (19%), NS4B protein gene: 184 substitutions (25%), NS5 protein gene: 560 substitutions (21%), and 3’UTR: 90 substitutions (20%)). Within the putative polyprotein precursor, 203 amino acid (aa) alterations (6%) are found (C protein: 12 substitutions (10%), preM-M protein: 7 substitutions (5%), E protein: 22 substitutions (5%), NS1 protein: 33 substitutions (10%), NS2A protein: 24 substitutions (11%), NS2B protein: 7 substitutions (6%), NS3 protein: 25 substitutions (5%), NS4A protein: 7 substitutions (6%), 2K protein: 1 substitution (5%), NS4B protein: 19 substitutions (8%), and NS5 protein: 46 substitutions (6%)). Due to this high level of sequence diversity, it is not possible to test the potential effect of each substitution in all combinations. As a result, within this study we only tested the effect (in a neuroinvasive WNV lineage 2 strain) of the nucleotide substitutions that have already been identified as virulence markers in WNV lineage 1 strains.

### 3.2. Rescue of Recombinant Viruses

In order to investigate whether molecular markers of virulence identified in lineage 1 WNV strains attenuate the Hungarian neuroinvasive lineage 2 WNV-578/10 strain *in vitro* or *in vivo*, infectious cDNA clones encoding each substitution of interest were constructed and transfected into BHK-21 cells. After transcription by CMV promoter, CPE had reached the maximum level at 72–96 h after transfection. Supernatants were harvested and passed on Vero E6 cells, which resulted in the generation of at least 6 log_10_ TCID_50_/mL virus stocks. When the titre did not reach the desired level, one more passage in Vero E6 cells was performed. All mutations of interest were verified by Sanger sequencing of each stock before *in vitro* and *in vivo* characterization. The NS4B102 mutant recombinant WNV was unable to multiply to a sufficient titre in BHK-21 cells nor after passage in Vero E6 cells, and this mutant clone was therefore excluded from the functional analyses. 

### 3.3. Full Genome Sequence Analysis of Recombinant Virus Stocks

To determine whether passaging the virus stocks led to the introduction of additional mutations, the full genome of the wild type and modified clone virus stocks were sequenced. One nucleotide alteration was found in addition to the mutated sites for three mutant clones, of which two out of three nt changes were silent: G to A on locus 627 of the NS1 clone, and A to G on locus 6768 of the NS4B38 clone. One nt alteration in the genome of the NS4B249 clone resulted in a Valine to Isoleucine change in the NS4B protein at locus 188 (G to A on locus 7480). The *in vitro* and *in vivo* characterization was subsequently carried out using the generated clones.

### 3.4. Multiplication Kinetics of the Recombinant Wild Type (WT) and Mutant WNVs in Cell Culture

**Figure 2:**
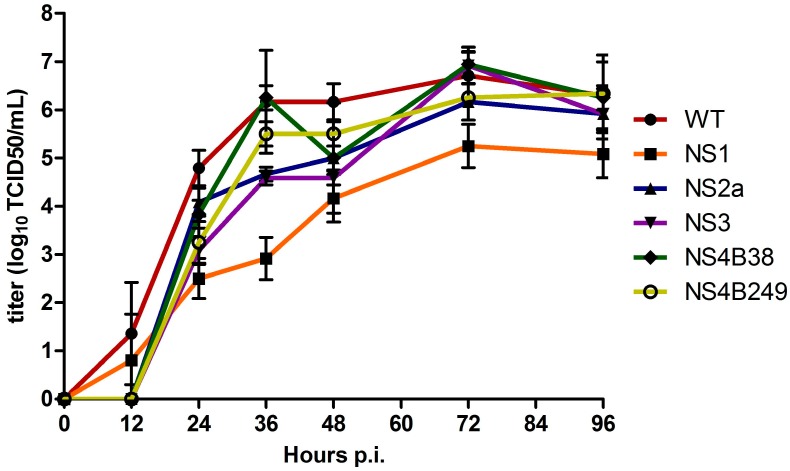
Growth kinetics of infectious virus of the wild type (WT) virus and mutant viruses after triplicate infection of Vero E6 cells at an MOI of 0.1. The titres are given as the mean (log10 TCID_50_/mL); error bars represent standard deviation.

The multiplication kinetics of the mutant WNVs were compared to those of the WT infectious clone-derived WNV-578/10 in Vero E6 cells infected in triplicate at an MOI of 0.1 at 37 °C, sampling every 12 hours. Overall, the infectious virus titers of the clones were significantly different at all time points between 12 h and 96 h p.i. (one way ANOVA; *p* < 0.02). More specifically, infectious virus titres for WT were significantly higher at 24 h p.i. compared to all the mutated viruses; NS1 (*p* = 0.005), NS2A, NS3, NS4B38, and NS4B249 (*p* = 0.03). Moreover, NS1 infectivity titres were found to be significantly lower compared to NS2A (*p* = 0.03) and NS4B38 (*p* = 0.03) (Figure 2). At 36 h p.i., WT infectivity titres were only significantly higher compared to NS1 (*p* = 0.002), NS2A and NS3 (both *p* = 0.02). At this time point, the difference in infectious virus titres between WT and NS1 was the largest, at approx. 2500-fold. In addition, NS1 titres remained significantly lower compared to all the other viral clones (all, *p* = 0.03). At 48 h p.i., WT infectious titres were still significantly higher compared to NS1 (*p* = 0.005), NS2A and NS3 (*p* = 0.03), while NS1 titres were only significantly lower compared to NS2A (*p* = 0.04) and NS4B249 (*p* = 0.03). At 72 and 96 h p.i., WT infectious titres remained only significantly higher compared to NS1 (*p* = 0.005; 0.02, respectively), while NS1 titres were significantly lower compared to all the other WNV strains at 72 h p.i. (*p* < 0.04), and compared to NS4B38 and NS4B249 at 96 h p.i. (*p* < 0.04). 

### 3.5. Quantification of Positive and Negative Strand RNA for WT and NS1 Mutant *in Vitro*

**Figure 3:**
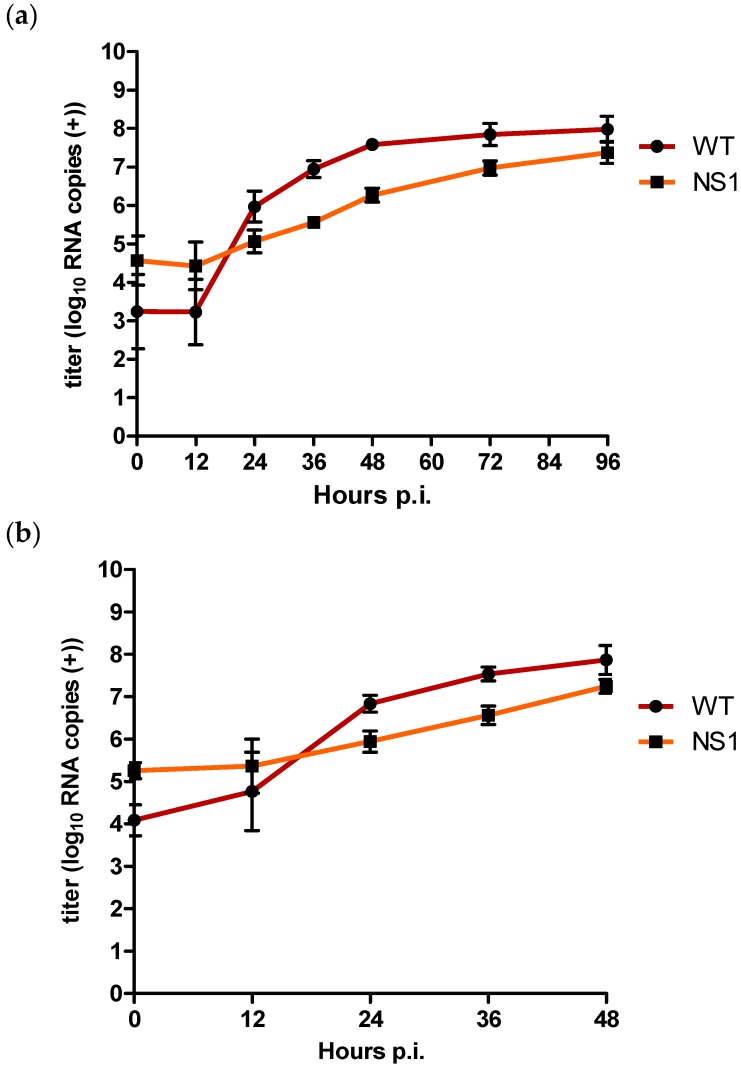

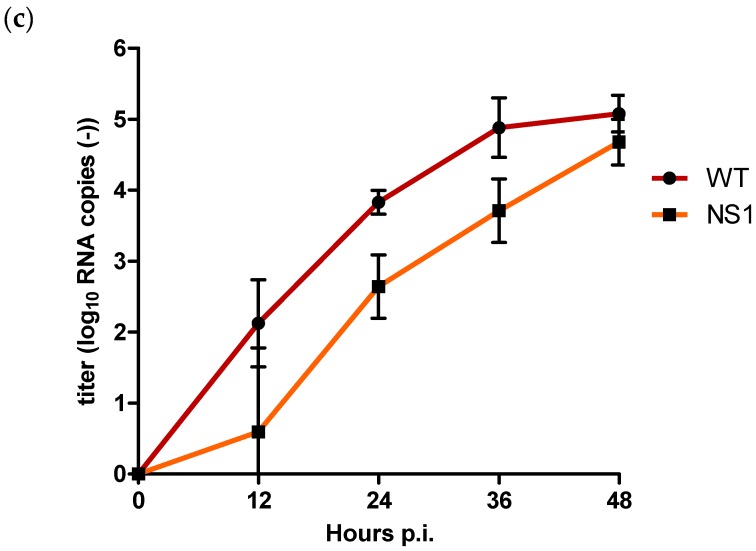
Quantification of (**a**) extracellular positive strand RNA (**b**) intracellular positive strand RNA, and (**c**) negative strand RNA for WT and NS1 mutant after triplicate infection of Vero E6 cells at an MOI of 0.1. Copy numbers are given as the mean of two independent experiments (log10 TCID_50_/mL); error bars represent standard deviation.

In order to assess whether the *in vitro* replication differences between the WT and NS1 mutant can be attributed to differences in positive and negative strand RNA synthesis, we determined the amount of positive and negative strand RNA copies in the supernatant and cells of the *in vitro* multiplication kinetics experiment using a strand-specific qRT-PCR assay [45]. The amount of positive strand present in the supernatant was more than 1 log_10_ higher for NS1 compared to WT at 12 h p.i., albeit not statistically significant (*p* > 0.05) (Figure 3a). At 24 h p.i., positive strand titres were approx. 0.9 log_10_ RNA copies higher for the WT compared to NS1 (*p* = 0.002). This difference increased to approx. 1.4 log_10_ RNA copies by 36 h p.i. (*p* = 0.002) and 1.3 log_10_ at 48 h p.i. (*p* = 0.002). At t = 72, titre differences had decreased to 0.8 log_10_ (*p* = 0.002) and at 96 h p.i. positive strand titres were only 0.6 log_10_ RNA copies higher for WT compared to NS1, although still significantly different (*p* = 0.04) (Figure 3a). 

Positive strand intracellular RNA titres were initially also higher for NS1 compared to WT for the first 12 h p.i. (Figure 3b). However, after 24 h p.i., WT positive strand RNA titres had become approx. 0.9 log_10_ RNA copies higher compared to NS1 (*p* = 0.002), with the most pronounced difference of approx. 1 log_10_ RNA copies after 36 h p.i. (*p* = 0.002). At 48 h p.i., positive strand RNA titres were still significantly higher for the WT compared to NS1 (*p* = 0.004) but the difference had decreased to approx. 0.6 log_10_ RNA copies (Figure 3b). 

For intracellular negative strand RNA copies, titres for NS1 were already significantly lower compared to the WT at 12 h p.i. (*p* = 0.02) (Figure 3c). NS1 negative strand RNA copies remained significantly lower compared to the WT with a difference of approx. 1.2 log_10_ RNA copies at both 24 and 36 h p.i. (*p* = 0.002). After 48 h p.i., titre differences decreased to approx. 0.4 log_10_ RNA copies, at which point the difference was no longer statistically significant (*p* = 0.06) (Figure 3c).

### 3.6. Mouse Neuroinvasiveness of the Different Mutant WNV Strains

**Figure 4:**
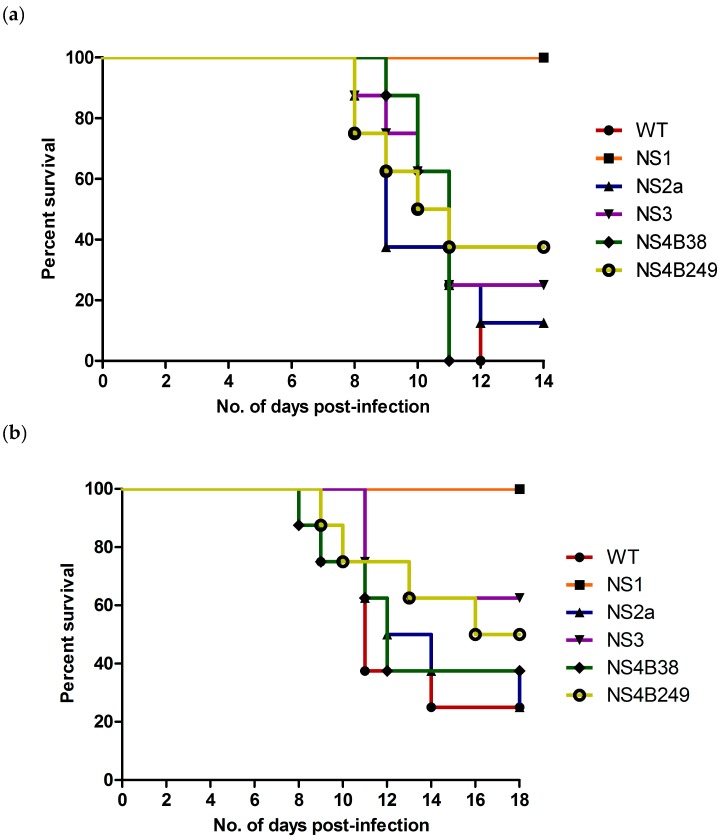
Survival of six-week-old female C57/Bl6 mice after i.p. inoculation, with (**a**) high dose (10^4^ TCID_50_) and (**b**) low dose (10^1^ TCID_50_) of recombinant WNVs.

The mouse neuroinvasive phenotype of each mutant WNV was examined following i.p. inoculation of six-week-old female C57BL/6 mice. Compared to the WT (100% and 75% mortality at high and low doses, respectively), only the NS1 mutant proved to be significantly attenuating since all mice infected with either the high or low dose of this virus survived the infection (0% mortality for both) (Table 3; Figure 4a,b). In contrast, mice infected with the highest dose of the other mutant strains experienced substantial mortality, with rates of 100% (8/8) for NS4B38, 88% (7/8) for NS2A, 75% (6/8) for NS3, and 63% (5/8) for NS4B249, respectively (Figure 4a). Statistical analysis confirmed the attenuation of the NS1 mutant, as significant differences were found between the survival curves of mice infected with the highest dose of NS1 as compared to the WT (*p*<0.0001), NS2A (*p* = 0.0004), NS3 (*p* = 0.003), NS4B38 (*p* = 0.0002) and NS4B249 (*p* = 0.008). On the other hand, the high dose survival curves 6of the other mutant WNVs showed no significant differences in comparison to the WT, or compared to each other. 

Mortality rates of mice infected with the lowest viral dose of the other mutant strains were 75% (6/8) for NS2A, 63% (5/8) for NS4B38, 50% (4/8) for NS4B249 and 38% (3/8) for NS3 (Table 3; Figure 4b). The lower dose survival curves of NS1-infected mice were also significantly different when compared to the WT (*p* = 0.002), NS2A (*p* = 0.002), NS4B38 (*p* = 0.009) and NS4B249 (*p* = 0.03). In contrast, the survival curves of the other mutants were not significantly different compared to the WT, or each other. 

### 3.7. Sequencing of Recombinant WNV in Organs of Euthanized and Survivor Mice

All mice euthanized due to illness were found to be positive for viral RNA in the brain (data not shown). To determine whether the mutant strains detected in the brains of mice euthanized due to illness after infection with the highest viral dose had retained the original mutation, viral RNA obtained from two mouse brains per group was sequenced. It was found that the consensus sequences of the virus strains present in the selected mouse brains still contained the original mutation and therefore had not reverted to the wild-type. Additionally, we also determined the presence of persisting virus in the brain and kidney of all mice that had survived infection (euthanized on day 14 p.i.) with the highest dose of the different mutant virus strains (NS1, n = 8; NS2A, n = 1; NS3, n = 2; and NS4B249, n = 3) using qRT-PCR. All brains positive for viral RNA were sequenced in order to check for the presence of the original mutation. Out of the eight mice that survived infection with NS1, only one mouse was found to be positive for virus in the brain, which had reverted to the wild-type. For NS2A, the one survivor mouse was found to be positive for viral RNA in the kidney, which still contained the original mutation. Out of the two mice that had survived infection with NS3, and out of the two mice surviving infection with NS4B249, only one mouse in each group was positive for viral RNA in the brain, which was also found to have retained the original mutation.

## 4. Discussion

The aim of this study was to generate a full-length infectious clone of the WNV lineage 2 strain 578/10 that has recently been detected in central Europe, in order to improve our understanding on the genetic background of WNV pathogenicity. Infectious cDNA clones are useful tools to investigate genetic determinants of flavivirus virulence, for studying its replication, creating subgenomic replicons, and using gene expression or gene of interest insertions [46,47,48]. Full-length infectious WNV clones are often constructed by fusion (stitching) polymerase chain reactions or plasmid-based methods [49,50,51,52]. Since assembly of full-length flavivirus clones in plasmid vectors have proven to be toxic, or unstable and deleterious for bacterial hosts, on several occasions [53,54,55,56], we cloned the full-length genome of WNV-578/10 using a bacterial artificial chromosome (BAC) [57]. This system contains the cytomegalovirus (CMV) immediate-early promoter that allows transcription of WNV RNA in the nucleus by cellular RNA polymerase II, eliminating the step of *in vitro* transcription.

WNVs belonging to lineage 2 were previously considered as agents of low pathogenicity [10]; however, numerous neuroinvasive and highly pathogenic members have recently been identified in horses and humans suffering from severe encephalitis in South Africa [58]. WNV strains belonging to lineage 2 have also been reported in Hungary and surrounding countries since its first proven appearance outside of Africa in 2004. During the last decade, several fatal cases among horses and humans were confirmed as West Nile encephalitis caused by these lineage 2 viruses in the Central European region [15,20]. The changing epidemiology and pathogenicity of WNV outbreaks in Europe highlight our need to further understand how Central European WNV lineage 2 strains differ in their capacity to cause severe disease compared to lineage 1 strains. Furthermore, this study may also provide invaluable information for the development of safe and efficacious vaccines. 

Several studies have identified and proven the role of genetic markers in the NS proteins of lineage 1 WNV strains in pathogenicity and virulence studies [24,26,30,31,33,34,35,36]. On the other hand, virulence markers of lineage 2 WNV strains have so far only been identified *in silico* by analyzing and comparing full genome sequences [37,38]. Thus far, it is known that flaviviral NS proteins are essential for virus replication; NS1, NS3, NS4A and NS4B can reorganize cellular membranes to generate virus-induced membrane structures (IMS) for site of replication [59]. Furthermore, NS2A [24], NS2B, NS3, NS4A [60], NS4B [61,62] and NS5 [63] play a role in virion assembly and evasion of host innate immune responses by blocking the interferon (IFN) signal transduction pathway [64,65,66].

The flavivirus nonstructural protein NS1 is a glycoprotein with three conserved *N*-linked glycosylation sites, and has an essential role in viral RNA replication. Normally, NS1 exists as a heat labile homodimer that associates with cellular organelle membranes and is transported to the cell surface [67,68]. Cell surface associated NS1 appears to have an immunomodulatory function via the decrease of the complement activation by different routes [69,70]. NS1 is also secreted by mammalian cells as a soluble hexamer [71,72]. 

Inclusion of the mutation P250L in a conserved region of the Kunjin virus NS1 gene has been shown to affect the structure of the polypeptide, resulting in the inhibition of dimer formation but still allowing its secretion in the monomeric form. The conformational change resulting from the P250L mutation may have led to the decreased levels of infectious virus titres observed in Vero cells in the early phase of replication (100-fold lower between 12–24 h p.i., compared to the WT); however, this difference gradually decreased and eventually disappeared by 48 h p.i. [73]. Similar to this study, our NS1 mutant also showed a 100-fold decrease in infectious virus; however, this was starting from 24 h p.i. lasting until 48 h p.i., after which the difference was approx. 10-fold until 96 h p.i. We investigated these observations further by quantifying the amount of positive and negative stranded RNA during the first 48 hours of replication in order to determine whether the mutation affected the replication process. We found that the NS1 mutant exhibited significantly slower replication as measured by an approx. 10-fold reduced amount of both negative and positive stranded RNA in the cells, as well as positive stranded RNA in the supernatant. 

A role for the importance of NS1 in the process of RNA synthesis has already been described in previous studies. Youn *et al*. found that WNV RNA lacking intact NS1 genes was efficiently translated but did not form canonical replication complexes early after infection, resulting in a failure to replicate viral RNA and consequently significantly lower amounts of positive and negative stranded RNA in the cells [74]. As we also found a reduced amount of negative and positive stranded RNA for the NS1 deletion mutant as compared to the WT during the first 48 hours of infection, it suggests that the P250L mutation potentially affects the activity or the stability of the NS1 protein for the formation of replication complexes during infection. 

In the study by Hall *et al.*, 10-fold more virus of the WNV-KUN P250L mutant was required to produce disease in mice. Our results, however, show that this mutation completely abolished the neuroinvasiveness of the lineage 2 WNV-578/10 strain, since no mice died after challenge with either the high or low dose of the virus. This means that in contrast to the Kunjin virus, neuroinvasiveness had decreased at least 10,000 fold for our mutant virus. It is possible that the different mouse model used in the aforementioned study (BALB/c mice) or the different age (18–20 days of age) of the mice explains the discrepancy between our results. However, it can also not be excluded that the silent mutation on locus 627 of the NS1 clone that we identified during full genome sequencing influenced the attenuated phenotype of this mutant. Furthermore, the presence of certain loci in the genome of the lineage 2 virus may have also augmented the attenuating effect of the P250L mutation observed in our study as compared to those involving lineage 1. Studies involving the introduction of this mutation into other, virulent lineage 1 or 2 WNV strains may provide more insight into the importance of this mutation.

Nonetheless, the significantly reduced replication of the NS1 mutant that we observed in cell culture may explain the reduced neuroinvasiveness observed in our study, as it may allow for early control of the immune system followed by elimination of the virus before it has reached the central nervous system. Even though only one mouse out of eight infected with the NS1 mutant was found to have virus in the brain at day 14 p.i. (albeit lacking the original mutation), we cannot rule out with certainty that the virus had still entered the brain in the majority of mice but had simply been cleared by day 14 p.i. due to its reduced neurovirulence. Future experiments involving intracranial injections of the NS1 mutant could confirm the reduced neurovirulent or neuroinvasive capacity of the virus.

NS2A is a small, hydrophobic, membrane-associated protein of WNV, believed to anchor the RNA replication complex by intercalating into the endoplasmic reticulum (ER) membrane [75]. The NS2A protein plays an important role in virus assembly and in the inhibition of the cellular antiviral response via the inhibition of IFN-beta promoter-driven transcription [32,76]. In one study, a NY99 mutant virus was less virulent in four-week-old Swiss-Webster outbred mice when the NS2A locus was derived from the Kunjin virus and harbored an introduced A30P mutation [26]. In contrast, Rossi *et al*. found only a slight decrease in virulence of the NS2A A30P mutant NY99 strain compared to the WT virus in five-week-old Swiss-Webster mice [25]. Our results are in line with the latter study, as we observed no significant differences in the replication kinetics of the mutant compared to the wild type virus, and no significant attenuation was detected in mice after challenge. 

The full length NS3 protein is a multifunctional enzyme (the N terminal residues encode trypsin-like serine protease, the C terminal residues encode RNA triphosphatase, NTPase and helicase) that possesses various activities in both viral polyprotein processing and RNA replication [77,78]. NS3 is suggested to be involved in virus assembly as well, but in cooperation with NS2A [79]. In corvids, the lineage 1 NY99 virus containing a proline to threonine substitution at the NS3-249 locus was particularly attenuating (100% to 12.5%), while the introduction of a proline at this site in a low virulent strain led to an increase in virulence (31% to 94%), likely related to an increased capacity of the virus to replicate in corvids [33]. This site therefore appears to be a key virulence determinant of the lineage 1 NY99 strain in corvids; however, the influence of this mutation in a mouse model has been minimal [80]. 

Genetic comparison of the goshawk-Hungary-2004 strain with the closely related lineage 2 Nea Santa-Greece-2010 identified an H249P mutation, which was speculated to play a role in the increased virulent phenotype of this Greek strain [18]. Interestingly, like the Greece-10 strain, our Hungarian lineage 2 WNV-578/10 strain also contains a proline at this position (data not shown). As a result, we investigated whether the NS3-249P mutation may contribute to increased virulence by introducing an NS3-P249H substitution and testing its attenuation in a mouse model. Here, this substitution proved to be slightly attenuating, but this was not statistically significant. Interestingly, infectious virus titres obtained in cell culture were found to be significantly lower for NS3 compared to the wild-type at three time-points. It should be noted, however, that this was also the case for several of the other mutants, such as NS2A, which had not shown any attenuation in our mouse model. It therefore appears that infectious virus titres *in vitro* do not correlate with attenuation *in vivo*. Indeed, in a study by Langevin *et al.*, despite the NS3-P249T mutation contributing to a 6 log_10_ PFU/mL lower titre in corvids, there were no significant differences observed in mean peak infectious viral titres on Vero cells at 37 °C for this mutant when compared to the NS3-249P virus [80]. As we were interested in examining the effect of the different mutations in a murine model, no thermosensitivity experiments at temperatures higher than 37 °C were undertaken for any of the clones; however, further studies testing the different mutants in cell culture at a higher temperature (41–44 °C) or in a corvid model might prove to be insightful as well, as in particular mutations at the NS3-249 locus seem to play an important role in the virulence of WNV in bird species. 

Flaviviral NS4B is a predominantly helical, hydrophobic, membrane-associated NS protein, which plays a crucial role in blocking host cell antiviral responses. It acts as an interferon antagonist, since expression of NS4B, contributed by the activity of NS4A and NS2A, strongly inhibits the IFN-induced signal transduction cascade by blocking STAT-1 phosphorylation [61]. Amino acid mutations in the coding region of NS4B can alter the inhibitory effect on interferon signaling [62]. 

The P38 residue of NS4B is predicted to localize to the junction of an ER-luminal region and a transmembrane domain. A study by Welte *et al*. found that the P38G mutation in the NS4B protein induced a lower level of viremia and no lethality in six- to ten-week-old C57BL/6 mice, while inducing higher type 1 IFNs and interleukin (IL)-1 as well as stronger effector and memory T cell responses [35]. A later study by Wicker *et al*. found the NS4B-P38G substitution to be associated with a temperature-sensitive phenotype of the lineage 1 NY99 strain, which involved a significant delay in multiplication in Vero cells at 41 °C, but not at 37 °C, as well as attenuation for neuroinvasiveness with an i.p. LD_50_ value of greater than 10,000 PFU in three- to four-week-old NIH Swiss mice. Importantly, however, two unexpected additional mutations were found at NS4B-T116I and NS3-N480H and actually none of the mutations alone were attenuating in mice [36]. In our study, the P38G mutation did not affect replication in Vero E6 cells and the virus was equally virulent in mice as the WT, where we used the same mouse strain and age as Welte *et al*. Furthermore, full genome sequencing revealed that the NS4B-T116I and NS3-N480H mutations were not present in our NS4B38 clone. Even though it is tempting to speculate that the P38G mutation may therefore not be so important for a lineage 2 WNV strain, we cannot exclude that the presence of other co-mutations, such as the silent mutation at the 6768 locus that we identified during full genome sequencing, or other mutations specifically present in the lineage 1 genetic backbone, are important for the attenuating effect of the NS4B-P38G mutation.

The C102S substitution of the NS4B protein in the NY99 strain has demonstrated thermosensitivity at 41 °C *in vitro* and was found to attenuate mouse neuroinvasiveness and neurovirulence [29]. The same substitution in the 578/10 construct dramatically reduced the replicative ability of the virus *in vitro* as such that no virus could be rescued, and therefore its further *in vitro* and *in vivo* effect could not be assessed. The hypothesized mechanism of C102S attenuation of the NY99 strain was a reduced ability in inhibiting the IFN signaling pathway [29]. However, the C102S mutant 578/10 clone was not able to replicate in BHK-21 and Vero E6 cells, even though the IFN-α and -β pathways are not functioning in these cell lines. Therefore, probably other factors contributed to the lethal effect of this substitution in the lineage 2 WNV strain. 

The NS4B-E249G mutation has been observed in several natural WNV isolates [81,82]. Furthermore, it was shown that a mutant lineage 1 WNV containing the E249G residue replicated at a lower level in C3H/He and BHK-21 cell cultures, but only slightly lower in Vero cell culture. In addition, the E249G mutant lineage 1 WNV was significantly attenuating in six-week-old C3H/HeN mice after footpad inoculation (100% mortality *vs*. 50%) [30]. On the other hand, a study by Rossi *et al.* reported that a WNV lineage 1 virus harboring the NS4B-E249G mutation demonstrated a WT phenotype with foci identical in size to the WT, as well as a similar LD_50_ value in i.p. inoculated five-week-old Swiss-Webster mice [25]. Our results are closer to those obtained by Rossi *et al.* [25], as the E249G mutant lineage 2 WNV propagated to similar titres in Vero E6 cells as the WT and showed reduced mortality in mice (63%) that was not significantly different. It cannot be excluded, however, that the non-conservative mutation found at NS4B188 played an important role in decreasing the attenuated phenotype exerted by the NS4B-E249G mutation.

In summary, our results have shown that in mammalian *in vitro* and *in vivo* models the NS1-P250L mutation contributed to significant attenuation of lineage 2 WNV, while the NS3-H249P and NS4B-E249G mutations conferred a partial, but not statistically significant reduction of virulence in mice. In contrast, the NS2A-A30P and NS4B-P38G were not attenuating at all *in vivo.* Even though it might be possible to conclude that the mutation at the NS1 locus could be an important marker of virulence in lineage 2 WNV strains, the fact that lineage 1 mutation studies as cited herein often showed varying results means that such results should still be addressed with caution. For example, mouse genotype and age may have an important influence on the outcome of mutational studies *in vivo*. The influence of other mutations in the particular genetic backbone used in certain studies may also play an important role. To be specific, the genetic backbone of the lineage 2 virus that we used may play a role in decreasing the attenuation of some of the markers that we have investigated, as well as in increasing the attenuation of the NS1 mutation. As a result, future studies investigating details of the NS1-P250L substitution should be performed in another vertebrate model, and also studies involving the introduction of this particular NS1 mutation into the genome of other lineage 2 viruses, or introducing the entire WNV-578 NS1 locus harboring this particular mutation into a lineage 1 strain, may prove to be insightful. 

The infectious clone described in this study provides a useful tool for testing the effect of hypothetical virulence marker loci in *in vitro* and *in vivo* model systems. Within the last decade, virulent lineage 2 WNV strains have emerged in Europe. For example, a descendant of the strain that had emerged in 2004 in Hungary caused an epidemic with an unforeseen large amount of neuroinvasive cases in Greece, in 2010. Another lineage 2 strain (Reb_VLG_07_H, GenBank accession FJ425721) emerged in the Volgograd region of Russia and caused human neurological cases [21,22]. In 2010, this strain also emerged in south-east Romania [23] and survived for at least three years [83]. As a result, genetic comparisons of different emergent isolates may help to identify and predict potential virulence markers. In this regard, our study has provided more insight into genetic markers that may contribute to the virulence of lineage 2 WNV strains.

## Figures and Tables

**Table 1:** List of primers used to generate overlapping fragments of the West Nile virus (WNV) genome in order to construct the full-length clone pWNV-578/10.

Primer code	Nucleotide sequence (5’→3’)	Nucleotide position (5’) *
1F†	*GAGCTCGTTTAGTGAACCGTA*GTAGTTCGCCTGTGTGAGC	1
1FRC†	GCTCACACAGGCGAACTACT*ACGGTTCACTAAACGAGCTC*	20
4750F	CACACACTATGGCACACCACTAAGG	4750
5426R	GACATCAGCCTGTGTGTGAGAGTGG	5426
8190F	AGACTGGCTGCACAGAGGACCTAAG	8190
9175R	GGTCTTCATTGAGGAATCCGAGAGC	9175
3’XbaSacIIR‡	*ATCCGCGGTCTAG*AGATCCTGTGTTCTAGCACCACAG	11026

† Bases in italics are part of cytomegalovirus (CMV) promoter. * Primer positions corresponding to the sequence of WNV-578/10 strain. ‡ Bases in italics are the extra restriction enzyme cleavage sites.

**Table 2:** List of primers used to insert point mutations into the genome of WNV-578/10.

Primer code	Nucleotide sequence (5’→3’)	Nucleotide Position (5’)*
NS1F	CATCACCTTGGCAGGA*C**T**C*AGAAGCAATCATAACAGGAGACC	3201
NS1R	GGTCTCCTGTTATGATTGCTTCT*G**A**G*TCCTGCCAAGGTGATG	3201
NS2AF	TTCGCAAGAGGTGGACG***C****CC*AAGATCAGCATTCCAGCTATCA	3596
NS2AR	TGATAGCTGGAATGCTGATCTT*GG**G***CGTCCACCTCTTGCGAA	3596
NS3F	GGTACCAAACCTCAGCAGTG*C**A**C*AGAGAGCACAGTGGAAATGA	5336
NS3R	TCATTTCCACTGTGCTCTCT*G**T**G*CACTGCTGAGGTTTGGTACC	5336
38NS4BF	TTCTTGCTTGATCTGCGG***GG****G*GCTACAGCATGGTCTCTCTAT	7012
38NS4BR	ATAGAGAGACCATGCTGTAGC*C**CC***CCGCAGATCAAGCAAGAA	7012
102NS4BF	TCAGCTCTCTTGCTGGCGGCCGGG*T**C**C*TGGGGCCAAGTGACCCTG ACTGTGACT	7198
102NS4BR	AGTCACAGTCAGGGTCACTTGGCCCCA*G**G**A*CCCGGCCGCCAGCAAGAGAGCTGA	7198
249NS4BF	GGACTCTCATCAAAAACATG*G**G**G*AAACCAGGCCTCAAGAG	7643
249NS4BR	CTCTTGAGGCCTGGTTT*C**C**C*CATGTTTTTGATGAGAGTCC	7643

* Primer positions corresponding to the sequence of WNV-578/10. Triplets in italics are the loci of mutations, modified nucleotides are in italics and bold.

**Table 3:** Mortality data of six-week-old female C57BL/6 mice inoculated i.p. with high and low doses of the WT and mutant lineage 2 WNVs.

Virus	Dose (TCID_50_)	Total mortality	Mortality (%)	Median day
WT	10^5	8/8	100	10.5
WT	10^1	6/8	75	11
NS1	10^4	0/8	0	NA
NS1	10^1	0/8	0	NA
NS2a	10^5.5	7/8	88	9
NS2a	10^1	6/8	75	11.5
NS3	10^3	6/8	75	10.5
NS3	10^0	3/8	38	11
NS4B38	10^4	8/8	100	11
NS4B38	10^0	5/8	63	11
NS4B249	10^4	5/8	63	9
NS4B249	10^1	4/8	50	11.5
